# Embryogenesis and Larval Biology of the Cold-Water Coral *Lophelia pertusa*

**DOI:** 10.1371/journal.pone.0102222

**Published:** 2014-07-16

**Authors:** Ann I. Larsson, Johanna Järnegren, Susanna M. Strömberg, Mikael P. Dahl, Tomas Lundälv, Sandra Brooke

**Affiliations:** 1 Department of Biological and Environmental Sciences, University of Gothenburg, Tjärnö, Sweden; 2 Norwegian Institute for Nature Research, Trondheim, Norway; 3 Sven Lovén Centre for Marine Sciences, University of Gothenburg, Tjärnö, Sweden; 4 Florida State University Coastal and Marine Laboratory, St Teresa, Florida, United States of America; Bangor University, United Kingdom

## Abstract

Cold-water coral reefs form spectacular and highly diverse ecosystems in the deep sea but little is known about reproduction, and virtually nothing about the larval biology in these corals. This study is based on data from two locations of the North East Atlantic and documents the first observations of embryogenesis and larval development in *Lophelia pertusa*, the most common framework-building cold-water scleractinian. Embryos developed in a more or less organized radial cleavage pattern from ∼160 µm large neutral or negatively buoyant eggs, to 120–270 µm long ciliated planulae. Embryogenesis was slow with cleavage occurring at intervals of 6–8 hours up to the 64-cell stage. Genetically characterized larvae were sexually derived, with maternal and paternal alleles present. Larvae were active swimmers (0.5 mm s^−1^) initially residing in the upper part of the water column, with bottom probing behavior starting 3–5 weeks after fertilization. Nematocysts had developed by day 30, coinciding with peak bottom-probing behavior, and possibly an indication that larvae are fully competent to settle at this time. Planulae survived for eight weeks under laboratory conditions, and preliminary results indicate that these planulae are planktotrophic. The late onset of competency and larval longevity suggests a high dispersal potential. Understanding larval biology and behavior is of paramount importance for biophysical modeling of larval dispersal, which forms the basis for predictions of connectivity among populations.

## Introduction

Cold-water corals form widespread and highly diverse ecosystems on continental shelves and slopes, ridges, seamounts and in fjords [1], [2], [3]. Framework-forming cold-water scleractinian corals can create some of the most spatially complex habitats found in the deep ocean, providing niches for many other species [2], [3], [4], . Due to their longevity and generally slow growth rates, cold-water scleractinians contain high-resolution records of long-term climate change [7], [8], and may be important speciation centers in the deep sea [9]. Of the seventeen species of framework-forming scleractinians, six are considered to be important reef-builders [3]. The most common of these is *Lophelia pertusa* (Linnaeus, 1758), which is known to create extensive reef tracts, particularly in the North Atlantic [1], [3].

Cold-water coral reefs are threatened directly by human activities such as bottom trawling, hydrocarbon drilling, seabed mining, and indirectly by climate change and ocean acidification. In several parts of the world marine protected areas (MPAs) have been established [10]. Understanding the reproductive biology and connectivity among populations is critical for studies of population dynamics, for management purposes and for the design of MPAs [11]. Despite their fundamental importance, very little is known about the reproduction and larval biology of cold-water scleractinian corals [12], [13]. Such information provides insight into population connectivity, recruitment rates and potential recovery from natural and anthropogenic damage. In contrast to shallow-water scleractinians, of which most are hermaphrodites [14], [15], most cold-water scleractinians (including *L. pertusa*) are gonochoric, which means they have separate sexes [12], . The dominant fertilization mechanism for reef-building corals in deep waters appears to be broadcast spawning, followed by a dispersive larval stage ([12] and references therein). In contrast, solitary deep sea and polar species can be gonochoristic brooders or sequential hermaphrodites [12], [17], [18]. Reproductive patterns within the scleractinia can be quite variable and complex. There appears to be some systematic trends in sexuality (i.e. gonochorism vs. hermaphroditism), but developmental mode (brooding vs. broadcast spawning) seem to be relatively plastic [13]. The embryogenesis, larval development and length of the pelagic larval phase are not known for most cold-water coral species. An exception is the facultatively zooxanthellate coral *Oculina varicosa* that forms extensive reef systems along the edge of the Florida shelf at 100 m depth [19]. This coral reproduces annually with broadcast spawning in late summer followed by embryogenesis and larval development in the water column [20], [21]. Histological studies show that *L. pertusa* also reproduces annually, releasing a single cohort of gametes [16], [22], [23]. Spawning begins in late-January in the NE Atlantic and late September in the western Atlantic. For the NE Atlantic populations, spawning occurred over a period of 6–8 weeks [16], rather than one highly synchronous short term event as seen in many tropical corals [15]. Waller and Tyler [22] postulated an onset of gametogenesis in July or August for *L. pertusa* from the Porcupine Seabight offshore Ireland while Brooke and Järnegren [16] found overlapping reproductive cycles with start of oogenesis in January in the Trondheim Fjord, Norway.

Reproduction in scleractinians is both sexual and asexual, and the relative contribution of each mode has profound effects on the genetic structure of populations [24]. Corals have many forms of asexual reproduction, e.g. fragmentation [25], polyp bail-out [26], [27], clonal planulae produced via parthenogenesis [28], [29], [30], [31], or production of genetically identical larvae due to indeterminate cleavage [32]. The latter three strategies allow long-range dispersal of clones, which may affect the genetic population structure substantially. To date, fragmentation is the only known mode of asexual reproduction in *L. pertusa* and can be highly important in certain populations [33].

Scleractinian corals (and anthozoans in general) produce uniformly ciliated planula larvae, usually elongated along the oral-aboral axis. The planulae may have a gastrovascular cavity and a mouth (depending on feeding mode), sometimes protractable [34]. Both lecithotrophic (non-feeding) [18] and planktotrophic (feeding) [35] feeding modes have been reported within scleractinia. Generally, anthozoan planulae are considered planktotrophic [34]; however, some species of stony corals release well-developed short-lived planulae or produce large oocytes which give rise to lecithotrophic larvae [15]. Waller and Tyler [22] suggested that *L. pertusa* larvae are lecithotrophic, based on their observed oocyte sizes.

Swimming planulae regulate their position in the water column in response to environmental cues such as light, gravity [21], [36], [37], and pressure changes [38]. At settlement, planulae have been found to respond to various water-soluble and insoluble chemical cues [39], [40], [41], [42], [43] and to water flow [44], [45]. Larval behavior as well as larval lifespan influence dispersal distance, which in turn affects gene flow between populations of conspecifics.

Studies of connectivity and gene flow between *L. pertusa* populations have depended on indirect methods that compare neutral genetic markers (a DNA sequence that does not affect adaptive fitness of an individual) among populations [33], [46], [47]. Studies of *L. pertusa* populations in the North Atlantic Ocean using genetic markers suggest that some gene flow is taking place across large geographic distances [46], [47]. This indicates that *L. pertusa* produces a relatively long-lived effectively dispersing larval stage, something which is further supported by the fact that it has colonized North Sea oil platforms far from known reef sites [48], [49], [50]. There is so far no information on whether all larvae are sexually produced or whether they can also be clonally derived. Studies of genetic markers are limited to reef localities that physically have been sampled and there is typically a lack of clear geographic population structure, a phenomenon termed ‘chaotic genetic patchiness’ [51]. This unpredictability means that the results cannot be used to extrapolate the degree of connectivity between any pair of un-sampled reef localities. Larvae are dispersed in the ocean via interplay between physical and biological processes, which are highly chaotic and nonlinear [52]. Euclidian distances is therefore a poor predictor of gene flow and studies that instead look at isolation by oceanographic distance seem to get a much higher degree of explanatory power (e.g. [53]. Our understanding of connectivity between *L. pertusa* reefs would benefit from combining genetic data with hydrodynamic modeling of larval dispersal. In such models, information about time of spawning, larval swimming behavior, time spent in the plankton (competency periods and longevity), and mortality rates are crucial inputs, although rarely available [54], [55].

This study presents the first documentation of embryogenesis and larval development in the cold-water coral *Lophelia pertusa*. Data are from two locations in the NE Atlantic and include observations at spawning and of larval behavior, swimming and longevity. We also used molecular markers to confirm the genetic origin of the *L. pertusa* larvae. These data are discussed in terms of consequences for larval dispersal and their applications for biophysical modeling of connectivity among populations.

## Materials and Methods

Successful fertilization of *L. pertusa* gametes and subsequent description of embryogenesis and larval development was achieved independently in two laboratories: the Norwegian Institute for Nature Research (NINA) in Trondheim, Norway, and the University of Gothenburg marine field station at Tjärnö, Sweden. For reasons of clarity the data from the two laboratories are presented both separately in the materials and methods, and results sections, but also analyzed in combination. Results presented are from 3 spawning seasons; 2009 and 2013 in Tjärnö, and 2009, 2012, and 2013 in Trondheim.

### Ethic statement

No specific permissions were required for sampling in the Trondheimsfjord. Collection of *L. pertusa* in Norway is regulated by the Law of Marine Resources (Havresursloven) under the jurisdiction of the Norwegian Fisheries Directorate. Norwegian scientists do not require specific permissions for these collections. We have also followed the Law of Management of Nature Diversity (Naturmangfoldloven) §6, ensuring the welfare of the collected organisms.

Coral sampling at the Tisler reef in Norway was conducted with permission from the Norwegian Fisheries Directorate and the Ytre Hvaler National Park Management. Export and import permits for the corals, following the Convention on International Trade in Endangered Species of Wild Fauna and Flora (CITES), were admitted by the Norwegian Environmental Agency and the Swedish Board of Agriculture. Law of Marine Resources (Havresursloven) and Law of Management of Nature Diversity (Naturmangfoldloven) §6 was followed during the collections.

### Trondheim

#### Collection and preparation

Fragments of *L. pertusa* were collected from the Trondheim Fjord using the Remotely Operated Vehicle (ROV) *Minerva*, from the Norwegian University of Science and Technology (NTNU). Samples were collected from a steep wall reef with minimum 20 m distance apart in a landing net, either individually or one of each color morph (orange and white) at a time to allow samples from different colonies to be distinguished from each other. For spawning purposes corals were collected from 10 colonies on January 16, 2012 and 7 colonies on June 27, 2012 at Stokkbergneset (63°28′10.6″N, 09°54′44.0″E, 123–430 m). These collections were supplemented with additional colonies collected in previous years and maintained in the lab. Once on deck, coral samples were placed individually in mesh-baskets and held in coolers containing deep-water from the Fjord for transport.

In a constant temperature room at Trondheim Biological Station (NTNU), sub-samples of approximately 20 polyps each were removed from each colony and placed together in five 100 L aquaria. Representatives of all individuals collected were therefore represented in each aquarium, maximizing the probability of reproductively mature colonies occurring in all aquaria. Samples from old colonies were randomly distributed among the five aquaria. Corals were maintained in flow-through sand-filtered fjord water pumped from 100 m depth in the Trondheim Fjord, at ambient fjord water temperature of 7–8°C, and fed weekly with copepods.

#### Embryonic and larval development

The aquaria were checked every 4–12 hours from early February to beginning of April for signs of spawning. When oocytes were observed in the aquaria, they were removed and observed under a Leica Wild M8 stereomicroscope to determine whether they were fertilized, then transferred to GF/C (1.2 µm pore size) filtered seawater in 2 L jars in which they were maintained in culture. Water was changed every second day. The diameters of 105 unpreserved oocytes were measured from the images taken under a Leica DM-IRB epi-fluorescent compound microscope, using ImageJ (version 1.34s) image analysis software [56] with Shape descriptor plug-in [57]. All cultures were maintained in the constant temperature room at 7–8°C.

To describe early development, embryos were examined every 6–12 hours, and ∼50 embryos were fixed in 10% formalin for later assessment of developmental stage. Additional sub-samples of each culture were taken of each developmental stage and fixed for scanning electron microscopy (SEM) in 2.5% glutaraldehyde for one hour before rinsing three times in Phosphate Buffered Saline (PBS) at pH 7.4. All samples were stored in PBS at 4°C until further processing. Prior to examination, the SEM specimens were post-fixed in 1% osmium tetroxide, dehydrated through increasing ethanol concentrations, critical point dried, mounted on a SEM microscope stub and sputter coated. These were then viewed and photographed under a Tescan Vega II SEM.

#### Larval size measurements

In 2012, sub-samples of larvae were taken for size measurements from two different cultures (spawned February 28 and March 7) at 7, 13, 20, 22 and 57 days post spawning. In 2013 cultures from spawning events on February 16, 21, and March 12 were measured 3, 5, 7, 8, 9, 12, and 13 days after spawning. All of these cultures may have been the product of more than one male and female, but since the fragments were mixed, the precise parentage for each culture is not known. Live larvae were observed under a stereo microscope, but dimensions (length and width) were measured using a Leica DM-IRB epi-flourescent microscope or a Motic BA310 LED light microscope, after brief fixation in 10% formalin. Measurements of the 20 and 22 day-old larvae were taken using larvae fixed in glutaraldehyde and stored in PBS. A total of 251 larvae were measured and added to the Tjärnö measurements.

### Tjärnö

#### Collection and preparation

A first collection of corals took place on September 15, 2008, from the Tisler Reef in the NE Skagerrak. Live corals were taken from two different colonies, 150 m apart at 100 m depth, using the ROV *Sperre SUB-Fighter 7500*. A large sample (male) was taken from a colony at the position 58°59′45.0″N, 10°58′02.6″E, and a smaller sample (female) from a colony at 58°59′40.4″N, 10°58′07.6″E. Both samples were of the white color morph. Shortly after collection, the colonies were divided into fragments varying in size from 11 to 39 polyps. The original purpose of this experiment was to assess effects of feeding on respiration and growth, and not for reproductive studies. Corals were kept in flow-through of sand-filtered seawater (32–34 psu and 8°C, with water intake at 45 m depth) and were fed 5 days a week with *Artemia salina* nauplii. On January 26 (2009) the coral pieces were placed in polycarbonate experimental chambers divided into eight 10 L aquaria, equipped with “Mississippi-type” paddle wheels for internal circulation [58]. In each aquaria, there were 5 pieces from the male colony and 1 piece from the female colony mounted in an upright position. Release of gametes resulting in production of larvae occurred on February 12 when chambers were sealed up for respiration measurements.

A second series of coral collection with the sole purpose of reproductive studies were conducted on December 21, 2012, at the Tisler Reef. Five samples from different colonies were taken from 87–105 m depth at positions between 58°59′41.4″N, 10°58′07.4″E and 58°59′39.5″N, 10°58′06.4″E by means of ROV (Ocean Modules V8 Sii), using a landing net. All collected coral material was of the white morph, and each sample was landed separately. Subsamples of individual polyps were taken for sex determination by dissection, and the samples were found to consist of one female and four males. Suitable fragments from each sample were subsequently divided over four 18 L tanks, with three male and four female fragments in each. During daytime the parental colonies were kept in flow-through seawater (5 µm filtered, Ametek polypropylene cartridge), and checked hourly as far as possible for signs of spawning. Flow-through was turned off overnight, relying on internal circulation governed by Mississippi paddles, to save gametes released during the night. Some female fragments were kept in a separate tank, in the hope of getting isolated oocytes to run a controlled fertilization experiment.

#### Embryonic and larval development

In 2009, embryos and larvae from the spawning at February 12 were kept at low densities (c. 1 embryo or larva ml^−1^) in sand-filtered seawater at 32–34 psu and 8°C. This corresponds to *in situ* water temperature during the spawning season (January–March) on the Tisler Reef, while *in situ* salinity ranges between 34 and 35 psu [59], [60]. Cultures were kept in small glass beakers (0.5–1 L), in the dark, and water was exchanged weekly. Starting one week after fertilization, subsamples of larvae were offered various substrates and settlement cues in the form of glass, plastics, stones, bivalve shells, Reef Construct (epoxy putty), coral skeleton (with and without tissue), and presence of live corals. Substrates were conditioned for 10 days, also offering a potential bacterial film.

In 2013, larvae were produced after a number of spawning events (between January 10 and February 28) when sperm and eggs were released more or less simultaneously in the tanks. On several occasions gamete release were observed and the exact timing known, and gametes were collected minutes after release. Each batch of embryos and larvae were kept in separate 2.5 L glass bowls, with change of water every 3–4 days, and followed as long as they remained alive. Embryos and larvae were kept in filtered seawater (5 µm, Ametek polypropylene cartridge), with the same temperature and salinity range as in 2009. On March 5, all remaining larvae (i.e. batch sp23jan) were fed with the fine fraction of mixed, lysed and centrifuged *Calanus* copepods while feeding trials under microscope were done on subsamples of larvae, testing pico-plankton, microalgae (*Isochrysis* sp. and *Rhodomonas* sp.), and a fine fraction of *Calanus*, respectively.

Embryos and larvae for scanning electron microscopy (SEM) were sampled on two occasions in 2009, at day six (n = 20) and day 31 (n = 15) after spawning. Larvae were fixed directly in 1% osmium tetroxide in filtered sea-water for one hour, rinsed in fresh water, dehydrated in a graded ethanol series, and preserved in 70% alcohol until further processing. Finally, larvae were critical point-dried and sputter-coated and examined under a Philips XL20 microscope.

#### Larval size measurements

Larvae were subsampled and photographed live under an Olympus BX51 light microscope equipped with a DP70 camera. A total of 13 larvae in 2009, and 238 in 2013, were measured using Image J (version 1.42a) with the aim to assess the effect of larval age on larval size and form. Length and width were measured, and volumes were estimated using the equation for calculating the volume of a prolate spheroid, with the major axis corresponding to the oral-aboral axis of the larvae. To avoid confounding of the possible increase in volume going from morula to blastula, or due to gastrulation, and to assess growth and shape-shifting during the actual larval phase, only larvae 10 days and older were included. Linear regressions (ordinary least squares) were performed to determine whether there was a significant relationship between larval age and **a)** length; **b)** length vs. width ratios; and **c)** volume, using RStudio (version 0.96.331, R version 2.15.1, 20129 and the car package). Length and width data from the Trondheim larvae (n = 251) were included in the analyses, and regressions were performed on larvae from the different origins separately. The aim with the volume calculations was to find out if larvae grew with time, and to avoid confounding due to differences among larval batches analyses were performed on different batches separately. Only batches that could be followed for a longer period (i.e. with repeated measurements from the same batch) were included in the analyses.

#### Larval swimming speed

Swimming speed was measured on three occasions at 7, 14, and 21 days after spawning in 2009. Larval swimming was measured using a dissecting microscope (Olympus SZX16) with an attached digital camera (Olympus DP70). A scale bar placed underneath the dish containing the larvae was used for distance calibration. Sequences of 3–12 seconds, when larvae swam horizontally and straight, were used to measure swimming speed. The distance travelled was measured on the computer screen using a transparent sheet. Five larvae were placed in each of a number of Petri dishes and were kept in 8°C and 11°C, respectively, with the higher temperature used only for day 21. One Petri dish at a time was placed under the dissecting microscope in a room temperature of 16°C and larval swimming was recorded during a two-minute period. To ensure that the temperature was kept close to constant during filming, the Petri dish was placed in a larger Petri dish with water of the same temperature. Usable sequences from the recordings resulted in swimming speed measurements at 8–9°C from 7 (n = 7), 14 (n = 11), and 21 (n = 9) days post-spawning, and also at 11–12°C from day 21 (n = 5).

The dependence of swimming speed on larval age was tested using a one-way ANOVA with larval age as a factor with 3 levels (7, 14 and 21 days) and larval swimming speed at 8–9°C as the dependent variable. The effect of temperature (8–9°C and 11–12°C) on swimming speed was tested using a one-way ANOVA on data from 21 days after spawning. Data in the ANOVA analyses were tested for homogeneity of variances using Cochran's test and a posteriori comparisons were made using Student-Newman-Keuls (SNK) procedure. A Type I error rate of 0.05 was used.

#### DNA analysis

Genomic DNA was extracted from coral polyp and larval tissue with the Viogene Blood and Tissue Genomic DNA Extraction Miniprep System, following the manufacturer's protocol. Both parental colonies sampled at the Tisler reef 2008, and sixteen planulae from the 2009 spawning, were genetically characterized using three microsatellite loci developed for *L. pertusa* [61]. Forward primers were 5′-labelled with fluorescent dyes (WellRED oligos, Proligo). Loci were amplified in 10 µL reactions using polymerase chain reaction (PCR) containing 30 ng µL^−1^ of template DNA, 0.05 U recombinant *Taq* DNA polymerase (TaKaRa TaqTM), 0.125 µM of forward and reverse primer, 10× buffer (Mg^2+^ free, pH 8.3, 1.5 mM) MgCl_2_, and 0.2 mM of each dNTP. A PCR amplification was performed under the following conditions: initial denaturation at 94°C (82 min), followed by 30 cycles: 94°C (30 s); 58°C (40 s); and 72°C (30 s), with a final extension at 72°C (10 min). Primer pairs were poolplexed and sized on a CEQ 8000 Genetic Analysis System.

## Results

Spawning occurred unpredictably in both laboratories, with no detectable diurnal or lunar pattern as well as repeatedly within short time-spans. Attempts to get isolated gametes for controlled fertilization experiments failed as oocytes were released by isolated females without sperm becoming available while oocytes were still viable. It was therefore challenging to obtain the synchronous cultures necessary to describe embryonic development in detail, and the timing of cleavage stages is proximal.

### Spawning observations

#### Trondheim

Spawning started in the middle of February and ended in the middle or end of March in all three spawning seasons covered. Fragments from the same colonies were observed to spawn occasionally during the entire spawning season, although the same individual polyp did not release gametes more than one day. Spawning occurred simultaneously in different aquaria. Usually oocytes and sperm were released in the same tank, resulting in almost 100% fertilization; however, on occasion spawning of males or females would occur alone. Oocytes were either ejected in mucus, which gave them positive buoyancy, or without mucus making them neutrally or negatively buoyant. Sperm release was visible as white clouds that quickly dispersed. Both males and females sometimes ejected their gametes forcibly, in ‘squirts’. The oocytes were spherical almost immediately after release with no visible germinal vesicle. Average oocyte diameter was 161±4.06 µm (mean ± SD, range: 151–177 µm, n = 105) for a batch of newly released oocytes. Oocytes spawned from orange females always contained orange pigment, whereas those from white females were always white. This suggests that whatever is conferring the orange coloration to the adult is being vertically transmitted to the oocytes.

#### Tjärnö

Release of gametes was observed on two dates in 2009. Sperm only was released on January 16, and on February 12 additional spawning from both male and female colonies produced larvae. The flow-through of water had been cut off in the afternoon of February 11, and the next day gametes were observed in 5 of 8 separate sealed up aquaria. Water from the aquaria was collected the following day (February 13) and the collected water contained sperm, oocytes and embryos in the 16-cell stage. Oocytes and embryos appeared neutrally buoyant as no sinking or floating was observed in still water.

The first spawning event in 2013 occurred on January 10, and gametes were released one to four times a day on 12 days until February 28. During each separate spawning event 2–4 polyps of each gender were actively releasing gametes. Only on one occasion up to 10 male polyps were active simultaneously, resulting in polyspermy. Some of the oocytes from this tank were completely covered in sperm, with tails radiating in all directions, and the entire batch of oocytes had to be discarded due to poor development. The same polyp was never seen releasing gametes during more than one day. Males were oozing sperm for 30–60 minutes while females forcibly released a big squirt of oocytes as they retracted, followed by a few smaller portions every 15–20 minutes. Newly released oocytes were neutrally buoyant, spreading out through the entire water mass. From the second day, however, embryos were more positively buoyant and found just beneath the surface. The newly released oocytes (or zygotes) measured 163±5.48 µm (mean ± SD, range: 127–178 µm, n = 333) in diameter and was often observed with sperm attached already minutes after release. Sperm tails measured 64±1.79 µm (n = 14), and the conical heads were approximately 5 µm long. Fertilization success was mostly close to 100%.

### Embryonic and larval development

#### Trondheim

**Figure 1:**
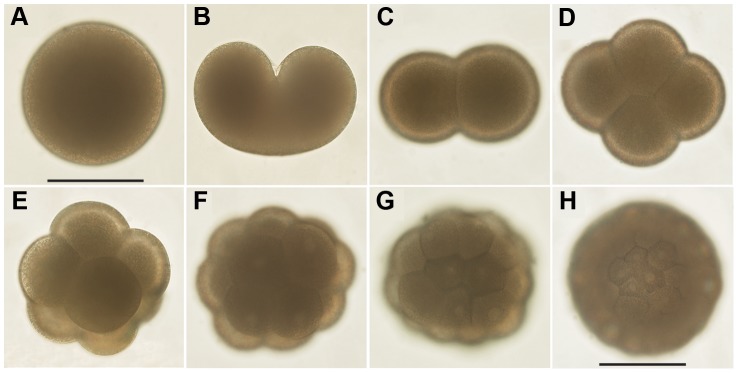
Compound microscope images of the embryo development of *Lophelia pertusa*. (**A**) oocyte; (**B**) 1^st^ cleavage; (**C**) 2-cell; (**D**) 4-cell; (**E**) 8-cell; (**F**) 16-cell morula; (**G**) 32-cell morula; (**H**) 64-cell blastula. First cleavage is equal and holoblastic, followed by radial cleavage. A slight shift of blastomeres sometimes made cleavage appear pseudo-spiral. Scale bar 100 µm.

**Figure 2:**
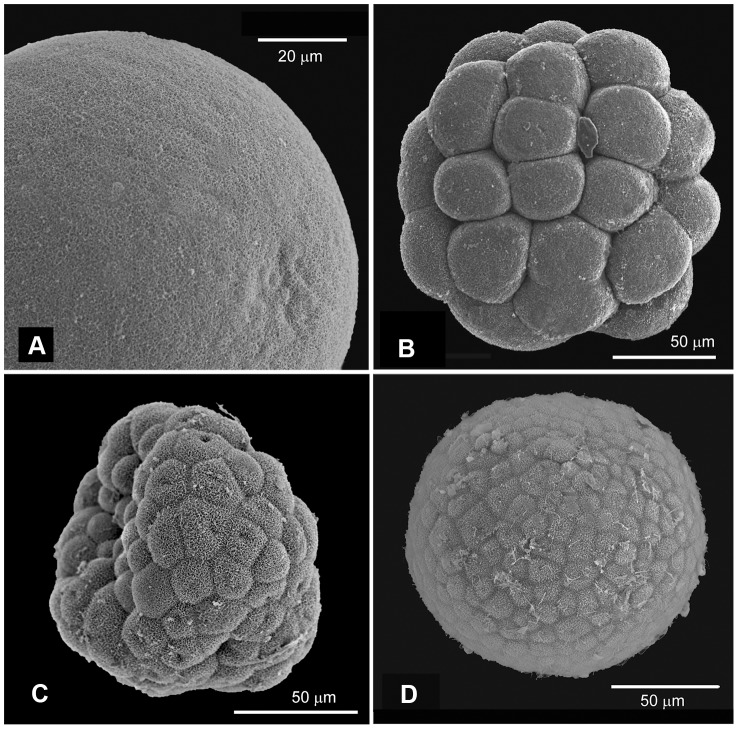
SEM images of *Lophelia pertusa* early development. (**A**) SEM image of an oocyte; (**B**) morula stage embryo; (**C**) Two days old embryo with indentation that possibly represents forming of the hollow blastula from morula stage; (**D**) late blastula showing sparse cilia coverage.

**Figure 3:**
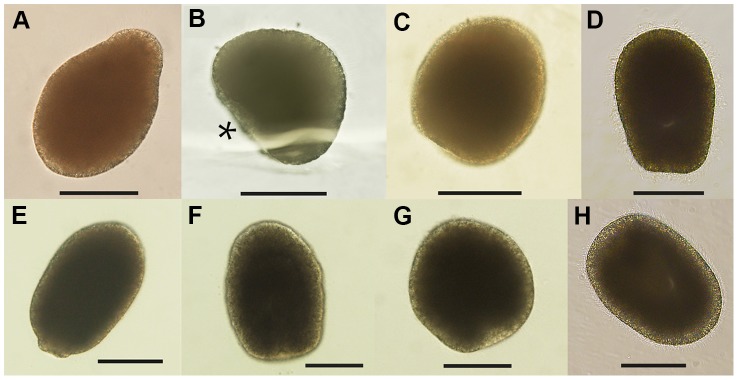
Light microscopy images of *Lophelia pertusa* embryos and larvae sampled at day 6 to 57 after spawning. Around day 6–7, embryos became piriform (**A**), followed by the formation of an indentation (*) indicating gastrulation sometime during day 6–8 (**B**). From day 10 larvae showed high plasticity, constantly shape-shifting (**C**–**H**). Scale bar = 100 µm. Larval age: **A** = 6; **B** = 7; **C** = 14; **D** = 22; **E** = 28; **F**–**G** = 44; **H** = 57 days old.

Cell division was equal and holoblastic and after the initial division, cleavage occurred at intervals of approximately 8 hours until the embryos reached the 64-cell stage (Fig. 1A–H). Cleavage was radial, although somewhat chaotic; i.e. the upper tier of blastomeres often shifted to lie in the cleavage furrows of the lower tier. Two days after spawning the developing embryos frequently were observed to have an irregular shape (Fig. 2C), after which they returned to a spherical shape. This possibly represents a change in shape as the embryo transitions from the morula stage to a hollow blastula. Ciliated blastulae formed approximately 3 days post-spawning, at which point they began slowly rotating (Fig. 2D). At 5 days they were still spherical but were spinning faster, corresponding with increased ciliation. At 7 days the larvae changed from a spherical shape and became more elongated. The larvae had some plasticity in shape, but were generally elongate spheroids, occasionally completely spherical (Fig. 3A–B). At this age, they began to swim rapidly in the normal planular fashion (aboral pole forward and spinning around the oral-aboral axis). At 12–14 days an oral pore became visible (Fig. 3C). As they matured, the larvae became densely ciliated, but with no apparent apical tuft. Once ciliated, the larvae remained at or close to the surface of the culture jars. At 14 days they were still in the upper part of the jars, but by 21 days post-spawning the larvae began to move towards the bottom of the culture jars. The larvae survived for a maximum of 57 days in the laboratory and during that time did not greatly increase in length (Fig. 3H).

#### Tjärnö

**Figure 4:**
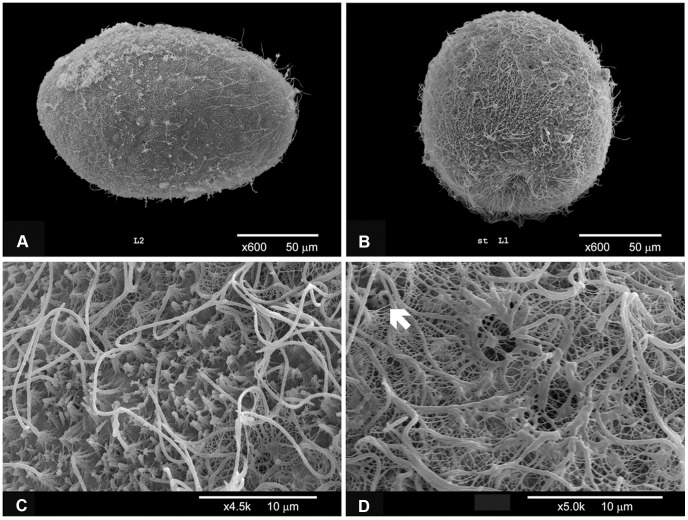
Scanning Electron Micrographs of *Lophelia pertusa* embryos and larvae. Image (**A**) shows a 6 days old ciliated embryo where each cell bears a cilium for locomotion. No oral pore or specialized structures are visible on the embryos at this stage; (**B**) A 4.5 weeks (31 days) old planula. The larva is fully and densely ciliated, and specialized structures are now visible. Larger pores are scattered over the surface, i.e. openings of mucus cells, and the oral pore facing downwards; (**C–D**) Close-ups of the body surface of 4.5 weeks old larvae; (**C**) ciliated ectoderm with mucus visible as web-like structures around the mucus cell openings; (**D**) mucus cell openings with webs of mucus. The white arrow marks out what appears to be a cnidocil, the trigger of a cnidocyst (a small bent cilium).

First cleavage started approximately 6 hours after gamete release and subsequent fertilization, and cleavage proceeded in a more or less well organized radial manner with 6 hour intervals until the 16-cell morula stage was reached after 24 hours (Fig. 1B–F). A blastula (64 cells or more) was formed after 48 hours (Fig. 1H), giving longer intervals between cleavages from 16 to 64-cell stage. First cilia appeared day 3, and embryos started twitching (Fig. 2D). Day 4 they were rotating, and by day 5 they were swimming spherical blastulae. Signs of gastrulation in the external morphology were not seen until day 6–8; the embryos first elongated into a piriform shape (Fig. 3A, Fig. 4A), then an indentation was formed (Fig. 3B). Embryos seemed to fuse up again, and an oral pore was not visible until day 13–14 (Fig. 3C). During gastrulation embryos were slowly rotating on the spot, after which they resumed swimming. Thus, they turned into a planula larva approximately day 7–9, after completed gastrulation. This process was not synchronized in the cultures; individual embryos were observed to gastrulate over the three days (day 6–8).

From day 10 and onwards larvae were plastic in shape and altered between elongate and more spherical morphology (Fig. 3C–H). Mature planulae were uniformly ciliated with cilia 10–40 µm long, and no apical tuft of longer cilia. The oral pore developed into a protractable mouth at about day 20 (Fig. 3D), and when larvae then were introduced to potential food items (pico-plankton, microalgae, and dissolved nutrients and small particles from mixed, lysed, and centrifuged *Calanus* copepods), two responses were observed: **a**) sweeping particles along the body sides through ciliary movements so that particles accumulated at the widely opened mouth (some particles were seen swept up into the mouth); **b**) while swimming, the larvae produced a mucus string in which particles adhered. No actual ingestion of mucus strings was observed in the limited number of observations done in this study. Nematocysts of three basic types were observed from day 30; atrichous isorhizas (spineless with tubule of uniform thickness), b-mastigophores (slender shaft without v-notch), and p-mastigophores (v-notched shaft). These were fired in contact with the edges of the coverslip, but not under the central part of the coverslip.

**Figure 5:**
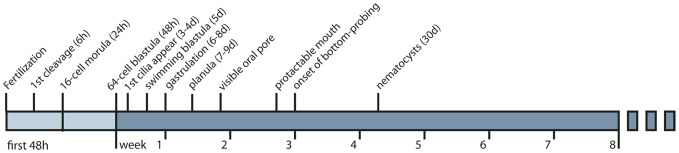
Timeline for the embryonic and larval development. Embryos reached the 64-cell blastula stage after 48 h. First cilia appeared day 3, and embryos were swimming blastulae day 5. During gastrulation (day 6–8) embryos were mainly still, rotating slowly around the oral-aboral axis, and resumed swimming after gastrulation was completed. An oral pore became visible when larvae were c. 2 weeks old, and the mouth became protractable and adjustable at c. 20 days. Bottom probing behavior was first observed when larvae were 3 weeks old and peaked at 4–5 weeks. Nematocysts appeared when larvae were 30 days old and if nematocysts are used for primary anchoring, this could indicate that larvae are competent to settle at this time. Larval longevity was c. 8 weeks, the longest lived larvae being 57 days old.

SEM images of 6 days old embryos (Fig. 4A) show that they at this stage were elongate and individual cells still visible, i.e. probably elongation prior to gastrulation as described above. Single cilia were developing from each cell and no oral pore or specialized structures were yet visible. The 4.5 week old larvae (Fig. 4B) were densely ciliated (due to the progress of cell-division and increasing number of ciliated cells) and had developed specialized structures; there were visible mucus cell openings surrounded by web-like structures (preparations of mucus, Fig. 4C–D); there was a structure that appeared to be a cnidocil (the trigger of cnidocytes, Fig. 4D); and an oral pore was clearly visible (Fig. 4B). A timeline summarizing the embryonic and larval development is presented in figure 5.

### Larval size measurements – combined data from Trondheim and Tjärnö

**Figure 6:**
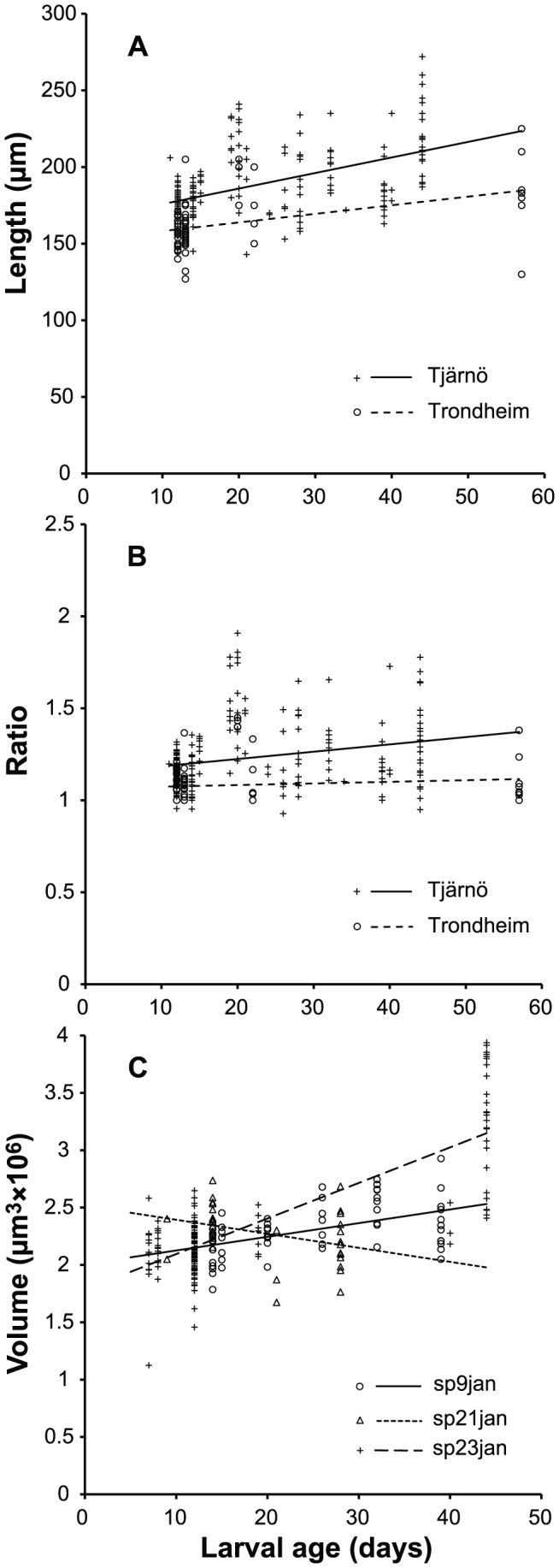
Size measurements of *Lophelia pertusa* larvae (≥10 days) from the Tjärnö and Trondheim laboratories. Larval length (**A**), length to width ratio (**B**), and volume of larvae from separate larval cultures at Tjärnö during the spawning 2013 (**C**). Lines are regression lines and the x-axis shows larval age in days after spawning. Batch sp23jan was fed on day 41.

Blastulae and larval lengths (measured day 3 through 57) ranged from 119 to 272 µm with an average of 173.4±21.05 µm (± SD, n = 506). Measured from day 10 and onwards, the length increased somewhat over time for both the Tjärnö planulae (Linear regression; 1.01, *r* ^2^ = 0.27, *F*_1,206_ = 78.4, *P*<0.001) and the Trondheim planulae (Linear regression; 0.56, *r* ^2^ = 0.21, *F*_1,85_ = 24.1, *P*<0.001; Fig. 6A). The length vs. width ratios (l/w) for larvae ≥10 days was an average of 1.19±0.18, i.e. larvae were in average slightly elongated along the oral-aboral axis. For the Tjärnö larvae, average l/w increased with time (Linear regression; 0.004, *r* ^2^ = 0.059, *F*_1,206_ = 13.9, *P*<0.001) whereas this was not the case for Trondheim larvae (Linear regression; 0.0009, *r* ^2^ = 0.0036, *F*_1,85_ = 1.31, *P* = 0.26). The variation was high, especially in larvae older than 15 days that ranged from spherical to almost twice as long as wide (Fig. 6B); the large variation being reflected in the very low *r* ^2^ values. Volume calculations revealed different trends for the three analyzed batches: i.e. larvae decreased in volume slightly with time in batch sp21jan (Linear regression; −0.015, *r* ^2^ = 0.17, *F*_1,29_ = 5.75, *P* = 0.023), increased slightly in batch sp9jan (Linear regression; 0.012, *r* ^2^ = 0.24, *F*_1,53_ = 17.1, *P* = 0.0001) and showed more pronounced increase in batch sp23jan (Linear regression; 0.032, *r* ^2^ = 0.65, *F*_1,110_ = 205, *P*<0.0001; Fig. 6C). These larval batches that could be followed for a longer period were all cultured in 5-µm filtered seawater without any nutrition added except on March 5 (day 41 for batch sp23jan, the only batch still remaining) when they were given potential food (fine fraction of mixed, lysed and centrifuged *Calanus*). The largest average volume of larvae was measured for batch sp23jan, three days after feeding (Fig. 6C), possibly as a result of food uptake.

### Larval swimming and behavior

The planulae swam in a spiral movement with the aboral pole forward, rotating clockwise around its longitudinal axis. During the first two weeks they were found exclusively in the upper half or one third of the jars, even when kept in the dark, indicating a negative geotactic behavior rather than a phototactic response. The first bottom-probing behavior was not observed until three weeks after fertilization and this behavior did not become more common among the larvae until 4–5 weeks after fertilization. Although larvae were offered various substrates and settlement cues, no permanent settlement or metamorphosis was detected. Some larvae were observed at the bottom, rotating around their axis while remaining on the same spot or slowly gliding along the bottom surface. Larvae attached temporarily to the bottom for seconds up to hours (slow water movement did not dislocate larvae) before swimming off returning into the water column. The last observation of a living larva was 48 days after fertilization in 2009, and in 2013 the separate batches of larvae lived for 6–8 weeks.

The swimming speed varied with larval developmental stage (Table 1). Late embryos/early planulae, seven days after fertilization, swam slower than 14 and 21 days old larvae in 8–9°C (one-way ANOVA, *F*_2,24_ = 13.7, *P*<0.0001). The swimming speed of the early stage was significantly lower than the swimming speed in both 14 and 21 days old larvae (SNK test, P<0.05). No difference in swimming speed between these two older larval stages was found. There was a trend of increased swimming speed with temperature in the 21 days old larvae (Table 1) but the difference was not statistically significant (one-way ANOVA, *F*_1,12_ = 1.30, *P* = 0.28).

**Table 1:** *Lophelia pertusa* larval swimming speed.

			Swimming speed (mm s^−1^)	
Age (days)	T (°C)	n	mean ± SD	min - max
7	8–9	7	0.23±0.07	0.15–0.36
14	8–9	11	0.51±0.09	0.36–0.67
21	8–9	9	0.53±0.19	0.29–0.86
21	11–12	5	0.65±0.19	0.49–0.93

The swimming speed was measured on embryo/larvae 7, 14, and 21 days after fertilization. Note that day 21 measurements were performed in two temperature ranges, 8–9°C and 11–12°C.

### DNA analysis

All sequenced larvae had one maternal and one paternal allele at the LpeA5 locus where both parental colonies were heterozygous (Table 2). The observed distribution of larval genotypes for the LpeA5 (χ^2^_2_ = 4.5, *P* = 0.21) and LpeA105 (χ^2^_2_ = 0.25, *P* = 0.62) loci did not differ from the expected ratio (1∶1) and conforms to Mendelian inheritance. Nearly all larvae were homozygous at the LpeC91 locus. Only two larvae inherited the 198 allele of the heterozygote parental genotype for the LpeC91 locus and did not conform to Mendelian distribution of alleles (χ^2^_2_ = 9.0, *P* = 0.003).

**Table 2:** Analysis of *Lophelia pertusa* larval genotypes.

Locus	Parental genotype		Number of observed larval genotypes				Expected ratio	?^2^_2_	*P*
LpeA105	274|274	286|295	9 (274|286)	7 (274|295)			1∶1	0.25	0.62
LpeA5	284|299	293|296	2 (284|293)	5 (284|296)	7 (293|299)	2 (296|299)	1∶1∶1∶1	4.5	0.21
LpeC91	202|202	198|202	14 (202|202)	2 (198|202)			1∶1	9.0	0.003

Results of Chi-square analyses of *Lophelia pertusa* larval genotypes observed at three loci. Genotypes are represented as six digit numbers, i.e. two alleles. The first and last three digits refer to the parental alleles (bp).

## Discussion

### Timing of spawning

Observations on gamete release in the laboratory (for both the Trondheim and Tjärnö corals) were consistent with the spawning period suggested for *Lophelia pertusa* in the NE Atlantic from histological studies [16], [22]. Coral fragments from both study areas released gametes over a period of approximately two months, with a shift in time between the two areas, i.e. spawning occurred in January to February in Tjärnö, and mid-February to late-March in Trondheim. These laboratory observations indicate that *L. pertusa* exhibits asynchronous spawning among individuals in the same population, and with slight geographical differences. Asynchronous spawning has also been documented in the laboratory for the shelf-edge scleractinian *Oculina varicosa* [20], for the temperate solitary coral *Caryophyllia smithii* [35], and for equatorial shallow corals [62]. Asynchronous or extended spawning may provide the population with some security against releasing gametes into unfavorable conditions or currents, and reduce the risk of losing an entire years reproductive output during a single spawning event [63]. This is particularly important in habitats where currents can be strong and variable, such as deep coral reefs; however, fertilization rates may be reduced with non-synchronous spawning and therefore, this strategy is not without risk [64], [65]. The observed polyspermy under laboratory conditions when 10 male polyps released sperm simultaneously (instead of the average 2–4 polyps) suggest high fertilization efficiency in *L. pertusa*, reducing the risk of too poor fertilization success rates.

### Embryogenesis, larval development and morphology

Spawned oocytes were ∼160 µm in diameter, which is slightly larger than oocytes documented from histology records [16], [22]. This is partly because histological processing causes shrinkage, and partly because the oocytes hydrate on release. White females produced white oocytes while orange females produced orange oocytes, suggesting that the coloration is transferred by vertical transmission, but it is unknown whether this is genetic or a result of pigmentation of other origin. Research on *L. pertusa* colonies from the Trondheim Fjord showed differences in microbial associates between the orange and white colonies [66]. The authors postulated that these differences may have ramifications for nutrition, which would be an interesting avenue to explore in the context of reproductive output.

Cnidarian embryos exhibit radial cleavage [67], [68] and although early cell divisions are radial, subsequent divisions do not follow a typical radial cleavage pattern. Various authors have described cnidarian embryonic development as appearing ‘pseudospiral’, ‘pseudoradial’ and ‘irregular’ [69], [70], [71]. In the sea pen *Ptilosarcus guernyi*, after each cleavage the blastomeres re-arranged to ‘resemble spiral cleavage’ [72]. The embryos of *L. pertusa* also showed a somewhat disorganized radial cleavage, as the upper tier of blastomeres sometimes shifted so that they were not lying directly over the lower ones, but were offset by approximately 45 degrees. Although not typical of radial cleavage, this pattern does not seem unusual for anthozoans [69], [70], [71], [72]. Radial cleavage has been considered a characteristic for deuterostomes [68] but is now considered being the ancestral mode of cleavage, while spiral cleavage is the derived mode with radial cleavage still at the deepest branches within spiralian clades [73], [74]. Cnidarian radial cleavage differs from the deuterostome (‘second mouth’) cleavage by being less organized, and in the polarity of the embryo, i.e. the first cleavage and subsequent gastrulation occurs at the animal pole [75], reflecting their simple bauplan with one opening, with the simultaneous function of mouth and anus.

Embryos remained approximately the same size as the oocytes during the early cleavage stages with cell division occurring every 6–8 hours during early development. The cleavage rate was slower than that observed in other coral species (0.5–1 hours) [21], [69], [71], [76], and the lower temperature of our larval cultures (corresponding to *in situ* temperature) was likely the reason for this slower development [21]. External morphological and behavioral changes indicating gastrulation is apparent not until day 6 to 8. The irregular shape of the 2-day old embryo (Fig. 2C), sometimes resembling an indentation as formed during gastrulation, could be a result of change of shape during formation of the hollow blastula from morula stage. The first observation of an oral pore not until two weeks post-fertilization (confirmed by SEM at 4.5 weeks) needs further attention. This is, however, congruent with gastrulation in the embryos of *Acropora millepora* in which the oral pore fuse up after gastrulation to produce a solid bilayered gastrula [77], the final mouth opening again at a later stage.

During the post-gastrulation period, larval morphology became increasingly complex with ciliation increasing from sparse coverage in late embryos (one week; Fig. 4A) to fully ciliated planulae at two weeks post-fertilization (as indicated by swimming speed). Specialized structures such as mucus cells and nematocysts developed between one and 4.5 weeks (Fig. 4). Nematocyst formation was confirmed to have occurred by day 30, when fired nematocysts were observed around larvae, and this could be interpreted as a sign of competence for settling. The Actinula larvae of *Tubularia mesembryanthemum* (Hydrozoa) have been shown to use nematocysts (atrichous isorhiza, also found here in *L. pertusa* planulae) for primary attachment during settling, before permanently cementing themselves to the substrate [78]. We observed temporary attachment with resumed swimming, previously also noted for other coral planulae [14]. Mucus cells were scattered over most of the larval surface, but mostly lacking around the oral pore. Larvae did not have the apical sensory organ at the aboral end (apical tuft) as observed in planulae from other cold-water coral [20], [21], [35] or anthozoan species [79], [80]. The *L. pertusa* planulae were plastic in shape, altering between spherical to more bullet-shaped. Both Tjärnö and Trondheim larvae were observed to attain an elongated piriform shape at about 1 week (Fig. 3A and 4A), just prior to the morphological changes that seemed to indicate gastrulation (during day 6–8).

From this study it seems that *L. pertusa* larvae may be planktotrophic rather than lecithotrophic as previously suggested by Waller and Tyler [22]. There are very few documented observations of coral larval feeding. One exception is the planktotrophic larvae of *C. smithii* which feed by producing ciliary currents causing food particles to pass from aboral to oral end, or by trailing mucus strings from the oral pole [35]. These planulae are initially of similar size as *L. pertusa* and *O. varicosa* (c. 150 µm) but grow to a length of 1 mm before settling. At the Tjärnö laboratory in 2013, larvae were kept in 5-µm filtered seawater except for one occasion when 41 day old larvae were given potential food particles. Both ciliary currents transporting food particles from aboral to oral end of the larva, and mucus trailing were then observed. The highest average volume of larvae was measured three days after feeding (day 44, Fig. 6C), possibly as a result of food uptake. For *O. varicosa* Brooke and Young [21] did not study possible feeding behavior, but adding of potential food in the cultures did not enhance larval development or survival.

### Larval swimming and behavior

The swimming behavior of *L. pertusa* planulae resembles that of other scleractinian larvae. Most coral larvae are observed to swim in a rotary clockwise or counter-clockwise manner around the oral-aboral axis and initially many of them are characterized by positive phototactic and/or negative geotactic responses [14]. Brooke and Young [21] studied phototactic and geotactic responses in *O. varicosa*. Early stage larvae swam towards the water surface responding to geotactic cues whereas later stage larvae, at the time benthic-probing behavior started, displayed negative phototactic behavior. In the present study, *L. pertusa* larvae appeared strongly negatively geotactic during the first weeks following fertilization. This may present a response to avoid the risk of being consumed by adult corals or other benthic predators [20], and/or be a response to reach the photic layer with its potentially higher quality and quantity of food. It was not until 3 weeks after fertilization that the first larva was seen to make contact with the bottom and this behavior did not become more common among the larvae until 4–5 weeks after fertilization.

The measured swimming velocity of *L. pertusa* planulae was twice as high for 14 and 21 days old larvae than for 7 days old embryos/planulae (Table 1). One probable explanation is that early stages are scarcely ciliated compared to fully developed planulae (Fig. 4). The swimming speeds of 2–3 weeks old fully developed *L. pertusa* larvae correspond very well to the swimming speeds measured by Brooke and Young [20], [21] on *O. varicosa* planulae at corresponding temperatures. The larvae of these two species are equal in size and are thereby equally affected by the viscosity of the water, i.e. they operate at similar low Reynolds numbers. In line with Brooke and Young [20], [21], we found that the average swimming velocity increased with temperature (Table 1) although the difference was not statistically significant due to low sample size and large variation in data in the three-week old larvae.

No larval settlement was observed in this study, although a variety of conditioned substrates and potential settlement cues were offered. Several cues have been identified for larval settlement and metamorphosis in tropical scleractinians, including chemical cues from coralline algae [40], coral skeleton [39] and microbial biofilms [41]. These water insoluble cues trigger larval decisions in the immediate vicinity of, and at impact with the substratum [81]. Recent experiments have shown that also water-soluble chemical cues stimulate settling in tropical corals [42], [43]. We did observe bottom probing in our jars, a behavior that may have been stimulated by water borne cues from e.g. the live coral fragments. The study by Lavaleye and Duineveld [82] indicates that larvae of cold-water corals respond to chemical and/or surface textural cues. On experimental panels deployed adjacent to thickets of *L. pertusa* and *Madrepora oculata*, they found coral spat exclusively on coral skeleton, rejecting ceramic tiles and oyster shells. Colonies of *L. pertusa* have been found growing on oilrigs [50], where no adult corals were present during initial larval settlement, therefore, the larvae must have additional cues than those associated with live corals or coral skeleton.

One potential settlement cue for *L. pertusa* larvae is water movement. Commonly *L. pertusa* reefs are associated with regions of elevated benthic flow with its associated high food availability [83], [84]. Many marine larvae of sessile benthic organisms do respond to flow during settlement and preferred flow regimes have been found to enhance subsequent growth and survival [85]. The response of coral larvae to flow is poorly investigated; however, Harii and Kayenne [44] found a species-specific relationship between flow speed and larval settlement rates in two coral species. Furthermore, studies on the temperate cup coral *Balanophyllia elegans* showed that water movement, and a suitable rock substrate, are equally critical factors for the settling of larvae [45]. Since a certain flow is needed for food delivery and reduced sedimentation load, this cue is potentially very important for *L. pertusa* larvae. This is one plausible explanation as to why no larvae settled despite the variety of substrates and cues offered. Another explanation could be that the larvae have to feed to gain competency for settlement and/or metamorphosis, and that the potential food available in the water after filtration was too scarce or of sub-optimal quality and that the potential food offered to the larvae were added to late. Further studies testing several factors, alone and in combination, are necessary to resolve the settling preferences and requirements for settling competency of *L. pertusa* larvae.

### Implications for larval dispersal and population dynamics

Larval dispersal in the ocean is a function of the interaction between physical and biological processes. The distance larvae are dispersed is governed by water flow and the amount of time larvae remain in the water column prior to settlement [52]. Observations showed that *L. pertusa* larvae actively swam upwards during the first weeks after fertilization, a behavior that will bring them out of the benthic boundary layer and promote advection by the stronger free stream currents. This, in combination with the three to five weeks time larvae spend in the water column before onset of bottom-probing behavior, imply that larvae have a great potential for dispersal. Since no settlement occurred in this study, the length of the larval competency period could not be deduced, but the maximum longevity of larvae was noted to be at least 8 weeks. This may be even longer *in situ*, especially if the larvae are feeding. The ability of *L. pertusa* to disperse far distances has earlier been inferred from observations of colonization of corals on oilrig constructions far from known reef sites. Several oil platforms with confirmed presence of *L. pertusa* are found in the northern North Sea [50]. Furthermore, genetic studies of the connectivity among *L. pertusa* populations in the NE Atlantic [46] and even across the North Atlantic Ocean [47] indicate that some gene flow occur also over large geographic distances.

The probability of *L. pertusa* planulae settling into the natal reef (i.e. individual smaller mound or reef site) should be rather small as deduced from the larval swimming behavior and the time spent in the water column. Living parts of the Tisler reef extend 1200 by 200 m laterally over a depth range of 70–150 m and is situated on top of a northwest-southeast oriented sill forming the connection between the Kosterfjord and the open Skagerrak [86]. The bathymetric situation forces the currents in a NW-SE direction and the direction of the residual currents usually remains constant for days to weeks [60], [86]. Tidal currents are small and tidally average residual flow strengths over the reef are usually in the range of 10–40 cm s^−1^, with peaks of over 60 cm s^−1^ [60], [86]. Under these circumstances larvae will be swept off the reef site within a few hours and the subsequent transport and dispersal during the following weeks before larvae are competent to settle will make chances returning to the natal reef quite low. Despite this, a recent study by Dahl *et al.* [33] of the genetic structure of reef complexes in the NE Skagerrak (Tisler reef and four adjacent reef complexes; maximum distance between any reef pair 35 km) indicated that some larval retention, or returning of larvae (local settlement), likely have occurred within the investigated area. For reefs located where other specific flow conditions prevail, e.g. tidal reversal [87] and typical flow patterns generated at seamounts [88], larvae should have greater potential of being retained on or return back to the natal reefs.

Vertical movement of larvae affects dispersal since the flow velocity and direction varies with depth. The variation with depth occurs in vertically stratified coastal waters but also in vertically mixed water columns where wind stress can establish an Ekman spiral [87]. Larvae of *L. pertusa* are active swimmers and with a swimming speed of 0.5–0.6 mm s^−1^ they are able to vertically migrate close to 50 m per day. A key question for biophysical modeling of *L. pertusa* larval dispersal is how far up the larvae will swim. Will they/are they able to cross density barriers and enter overlaying water masses of a different salinity, temperature and flow regime where they are transported for a period before onset of bottom probing? CTD-data recorded in the vicinity of the Tisler reef show that there is a transition from deeper warmer and more saline water to overlaying less saline and colder water at 20–40 m depth during the spawning season (Tomas Lundälv, pers. obs.). Dullo et al. [89] found that the distribution of *L. pertusa* colonies in two shelf regions of the NE Atlantic was strongly correlated to a narrow range of water density. One of several hypothetical explanations they presented was that *L. pertusa* larvae could have a neutral density in the density envelope where the adults are found. However, since *L. pertusa* larvae swim and are able to regulate their position in the water column this will only hold true if the larvae are not willing and/or able to cross into water masses of different physical properties. Future studies of larval behavior and possibly field observations will hopefully answer this question and give insight to the depth distribution of larvae during the dispersal stage.

Genetic assessment with codominant molecular markers has previously been done on corals when either one [28], [29] or both [90], [91], [92] parental genotypes were known, to confirm whether Mendelian inheritance of alleles occurred or not. All sixteen larvae in the present study were found to be sexually derived and Mendelian segregation could be confirmed at two loci out of three. The deviation from the expected ratio on the third loci may be due to the relatively low sample size, hence chance alone. Confirmation of non-Mendelian inheritance of sexual progeny from diploid organisms has been found with microsatellite markers [93], [94]. Controlled crosses of known genotypes are suggested to be standard part of marker development when possible [95] to assure proper analysis for detecting demographic patterns, including evaluation of contribution of asexual reproduction and inferring population connectivity. This has not been possible for *L. pertusa* previously since this is the first time larvae have been observed for the species. However, making this test in retrospect confirms the findings when these markers have been applied. If asexually produced larvae would have been encountered, this could have affected the interpretation of previous results from both small and large-scale genetic structure studies that have been conducted on *L. pertusa* [33], [46], [47]. Clonal planulae (produced via parthogenesis) have up to recently been associated with brooding species only [28], [29], [30], [31]. Heyward and Negri [32], however, describe the possible importance of indeterminate cleavage in the development of planktonic clonal coral larvae. During maintenance of our larval cultures we could see some indications of blastomere splitting in early embryonic development, with continued development to functional (but smaller) larvae. No formal experiments were run to explicitly test this, however, now that successful breeding of *L. pertusa* larvae has been accomplished there is opportunity for further testing.

The type of information on *L. pertusa* larval biology presented in this study can be integrated into hydrodynamic models to predict population connectivity [96], [97]. Such predictions are particularly important for understanding population dynamics at different spatial scales and to assess potential resilience from large-scale mortality events. Knowledge about connectivity is furthermore critical for management purposes and for the design of marine protected areas [11].
